# Changes in Gastrointestinal Microbiome Composition in PD: A Pivotal Role of Covariates

**DOI:** 10.3389/fneur.2020.01041

**Published:** 2020-09-23

**Authors:** Alexandra Cosma-Grigorov, Holger Meixner, Anne Mrochen, Stefan Wirtz, Jürgen Winkler, Franz Marxreiter

**Affiliations:** ^1^Department of Molecular Neurology, Friedrich-Alexander-Universität Erlangen-Nürnberg (FAU), University Hospital Erlangen, Erlangen, Germany; ^2^Department of Neurology and Neuroscience, Albert-Ludwigs-University Medical Center Freiburg, Freiburg, Germany; ^3^Department of Neurology, FAU, University Hospital Erlangen, Erlangen, Germany; ^4^Department of Medicine 1, FAU, University Hospital Erlangen, Erlangen, Germany

**Keywords:** gut, microbiome, Parkinson's disease, constipation, non-motor symptoms, biomarker, covariates

## Abstract

Altered gut microbiota may trigger or accelerate alpha-synuclein aggregation in the enteric nervous system in Parkinson's disease (PD). While several previous studies observed gut microbiota alterations in PD, findings like diversity indices, and altered bacterial taxa itself show a considerable heterogeneity across studies. We recruited 179 participants, of whom 101 fulfilled stringent inclusion criteria. Subsequently, the composition of the gut microbiota in 71 PD patients and 30 healthy controls was analyzed, sequencing V3–V4 regions of the bacterial 16S ribosomal RNA gene in fecal samples. Our goal was (1) to evaluate whether gut microbiota are altered in a southern German PD cohort, (2) to delineate the influence of disease duration, stage, and motor impairment, and (3) to investigate the influence of PD associated covariates like constipation and coffee consumption. Aiming to control for a large variety of covariates, strict inclusion criteria were applied. Finally, propensity score matching was performed to correct for, and to delineate the effect of remaining covariates (non-motor symptom (NMS) burden, constipation, and coffee consumption) on microbiota composition. Prior to matching altered abundances of distinct bacterial classes, orders, families, and genera were observed. Both, disease duration, and stage influenced microbiome composition. Interestingly, levodopa equivalent dose influenced the correlation of taxa with disease duration, while motor impairment did not. Applying different statistical tests, and after propensity score matching to control for NMS burden, constipation and coffee consumption, *Faecalibacterium* and *Ruminococcus* were most consistently reduced in PD compared to controls. Taken together, similar to previous studies, alterations of several taxa were observed in PD. Yet, further controlling for PD associated covariates such as constipation and coffee consumption revealed a pivotal role of these covariates. Our data highlight the impact of these PD associated covariates on microbiota composition in PD. This suggests that altered microbiota may mediate the protective effect of i.e., coffee consumption and the negative effect of constipation in PD.

## Introduction

In recent years, the perception of Parkinson's disease (PD) has transformed from a movement disorder into that of a multisystem disorder of the central nervous system (CNS) (1). Non-motor symptoms (NMS) such as constipation, REM-sleep-behavior disorder (RBD), and hyposmia have been recognized as surrogate parameters, often preceding motor symptoms by years. NMS are used to define prodromal stages of PD for research purposes (2). Among them, gastrointestinal impairment proceeds motor symptoms potentially by decades (3) and deteriorates with disease progression (4). Accordingly, accumulating evidence suggests that the enteric nervous system (ENS) may be the initial site of alpha-synuclein aggregation, eventually representing the onset of PD. However, alpha-synuclein positivity is observed in colon tissue of control subjects with prevalences ranging from 26% to even 91% across different studies (5, 6). Due to the ENS's close proximity to the gut lumen, and the huge surface of the mucosal barrier to the gut, significant interactions with the residing microbiota occur. This may be particularly relevant in altering or triggering neurodegenerative processes in PD, as it has already been proposed for a variety of other CNS diseases (7, 8). Two pathways by which the microbiota may induce neurodegeneration in PD have been proposed. One hypothesis is that dysbiotic gut microbial composition leads to increased gut leakiness. Consecutive gut inflammation fuels the accumulation of alpha-synuclein in the ENS, which is then propagated via a prion-like mechanism across the vagal nerve to the CNS (7). This propagation has recently been demonstrated in two murine studies (9, 10). This hypothesis is in line with the Braak Stages (11). In this scenario, a “disease causing” or “risk” microbiome, present already in prodromal and early disease stages may be a causative factor. A second hypothesis is that the gut microbiota's interaction with the ENS immune system may shape Th17 response, which has been shown for multiple sclerosis (MS) (12), leading to increased inflammation fueling PD progression (13).

Several cross-sectional studies observed an altered gut microbiota in PD patients (14), suggesting that it may serve as a biomarker for premotor PD or constitute a trigger for disease progression. Changes in microbial communities are also observed in atypical parkinsonism (MSA and PSP) (15). One study found gut microbiota alterations in subjects suffering from the PD prodrome iRBD compared to healthy controls (16). Yet, alterations in microbiota composition vary between studies. Also, it is currently not well-understood how disease duration and stage, and motor impairment affect microbiota composition in PD. Summaries of observed changes and the methods used in previous studies have been published, recently (14, 17). While dysbiosis has been shown in all studies, a consistent picture of a “PD specific microbiome composition” is debatable.

Microbiota are influenced by a vast variety of covariates such as regional lifestyle factors like nutrition (18), and importantly constipation, a prominent NMS in PD which worsens during disease progression (4). In addition, the interaction between gut microbiota and dopaminergic medication as well as anticholinergics has recently been recognized. Significant differences in gut microbiota as a result of treatment with catechyl-o-methyl transferase inhibitors (iCOMT) and anticholinergics as well as metabolism of levodopa by gut microbiota have been shown (19, 20). These covariates along with different methodological approaches used across studies may profoundly influence results. Moreover, consensus criteria on how to conduct a microbiome study are already 5 years old (21). Thus, there is currently no up-to-date consensus in this rapidly developing field that standardizes methods and provides guidelines on how to control for technological and procedural covariates.

This study aimed to provide further evidence for altered gut microbiota in PD in a southern German cohort, focusing on the effect of disease stage and duration, and in particular the influence of PD associated covariates like NMS burden, constipation, and coffee consumption.

## Materials and Methods

### Study Population and Inclusion Criteria

This observational study consisted of 179 participants, in total. All participants were recruited in the outpatient clinic of the Department of Molecular Neurology of the University Hospital Erlangen. We recruited patients' spouses and relatives as controls. The study was approved by the local ethics commission (No. 284_16 B), and all participants gave written informed consent.

PD was diagnosed by a movement disorder specialist according to the MDS clinical diagnostic criteria for PD (22). Motor symptoms were measured using part III of the Unified Parkinson's Disease Rating Scale (UPDRS III), and disease staging followed the original Hoehn + Yahr scale (H&Y) (23, 24). NMS burden was assessed using the Non-Motor Symptoms Scale (25). Constipation and associated symptoms were measured using the Cleveland Clinic Constipation Score (Wexner Constipation Score) (26). Signs of iRBD were analyzed using the REM Sleep Behavior Disorder Screening Questionnaire (RBDSQ) (27). Screening for depressive symptoms was performed using the 30-Item Geriatric Depression Scale (GDS30) (27, 28). Control subjects had no clinical signs of neurodegenerative disorders or symptoms suggestive of prodromal PD (2). Patients with monogenic forms of PD or more than one relative with PD were excluded, as were patients in H&Y stage 5.

Since no definitive consensus on inclusion and exclusion criteria in microbiome studies exists, we designed the study protocol to cover a wide range of the exclusion criteria used in a previous study in PD (29), and followed previously suggested consensus criteria (21). Briefly, for all participants, active or persistent primary disease of gastrointestinal tract, previous abdominal or anorectal surgery, previous vagotomy, antibiotic treatment within the last month and regular use of opioids were considered exclusion criteria. Inclusion and exclusion criteria were assessed by a self-report questionnaire, including a variety of nutritional factors, and in particular, coffee consumption on ordinal scale variables. Physical activity was measured as hrs per week of easy, moderate, and demanding physical activity. In case of unprecise reports, clarification was achieved by either telephone or interview during a regular outpatient visit. An English version of the questionnaire is provided within the Supplementary Material. A detailed table of all exclusion criteria is provided in Supplementary Table 1.

### Analysis of Fecal Microbiota

All participants collected fecal samples in a DNA stabilizing solution (Stool Collection Tubes with Stool DNA Stabilizer; Stratec®, Stratec Molecular, Berlin, Germany) at home, following a precise protocol provided with the Stool collection tubes, including the use of a feces collection paper in order to minimize contamination. Samples had to be transferred to the laboratory within 3 days and were immediately stored at −80°C upon arrival until further processing. Thus, only samplers arriving within a time frame of 72 h after collection were included. It is important to note that DNA stabilizing solutions are able to preserve microbiome profiles for up to 7 days at room temperature (30).

### DNA Extraction, PCR and Sequencing

Bacterial genomic DNA from stools was isolated using PSP® Spin Stool DNA *Plus* Kit (Stratec Molecular, Berlin, Germany) including a bead-beating step, following the manufacturers protocol. DNA was subsequently quantified using a Qbit device (Thermo Fisher Scientific). The V3+4 region of the 16S rRNA gene was amplified using 10 ng of bacterial template DNA with degenerate region-specific primers (341F: 5′-ACTCCTACGGGAGGCAGCAG-3′; 806R: 5′-GGACTACHVGGGTWTCTAAT-3′) containing barcodes and Illumina flow cell adaptor sequences (31) in a reaction consisting of 25 PCR cycles (98°C 15 s, 58°C 20 s, 72°C 40 s) using the NEBNext Ultra II Q5 Master Mix (New England Biolabs, Ipswich, MA). Amplicons were purified with Agencourt AMPure XP Beads (Beckmann Coulter, Brea, CA), normalized and pooled before sequencing on an Illumina MiSeq device using a 600-cycle paired-end kit and the standard Illumina HP10 and HP11 sequencing primers. For bioinformatic processing, the terminal 15 bases of both forward and reverse reads were removed before merging and quality filtering using the fastq_mergepairs and fastq_filter_options from Usearch 10 (32). Subsequently, merged fastq files were demultiplexed and trimmed using Cutadapt (33). 16S taxonomic sequence clustering (ASV table generation) and classification was performed with the Unoise3 (34) and Sintax (35) algorithms within Usearch using the greengenes 16S rRNA database v13.5.

### Statistical Analysis

Statistical analysis of clinical variables was performed using SPSS® Statistics Version 21 (IBM Corp.). Normal distribution was assessed by a Kolmogorov-Smirnov test. Group differences of normally distributed variables were analyzed using a *t*-test, non-parametric variables were assessed by a Mann-Whitney *U*-test. Differences in binary variables were tested using Fisher's exact test. Differences regarding all other categorical variables were tested using *z*-test followed by Bonferroni correction. For multiple comparisons of normally distributed variables, we used ANOVA with Bonferroni correction. For non-parametric variables, a Kruskal Wallis test followed by Bonferroni correction was used.

Statistical analysis of microbiome data was performed using marker-gene data profiling in MicrobiomeAnalyst (Xia Lab, McGill University, Quebec, Canada) (36). We followed recently published methods on data processing, normalization, and profiling (37). A low count filter was used to filter all features with <4 counts in at least 20% of values. Features with <10% variance, based on the inter-quartile rank, between experimental conditions (PD vs. controls, PD vs. H&Y) were filtered using a low variance filter. All samples were rarefied to even sequencing depth using the minimum library size (3,537 reads). Finally, for data scaling, total sum scaling was applied.

Diversity measures to calculate alpha diversity were Chao1 (richness of a group) as well as Shannon, and Simpson (richness and evenness of a group) using a *t*-test. In order to calculate beta diversity, distance methods used were Bray-Curtis Index using PERMANOVA. Since results based on sequencing of the V3 and V4 regions of the bacterial 16S ribosomal RNA gene are not reliable for species level (38), we did not pursue analysis for this taxonomic level. Moreover, altered bacterial taxa with <10 absolute counts were considered as not biologically relevant and excluded.

To analyze differences in the abundances of individual taxa on five major taxonomic levels (class, order, family, and genus) between controls vs. PD, or controls vs. disease stages H&Y1, H&Y2, H&Y3, H&Y4, we first performed classical group comparison using a Kruskal Wallis test in MicrobiomeAnalyst. In addition, a Wilcoxon signed rank test was used to assess differences between PD and controls, and results are displayed using heat tree analysis (39). To further delineate, which genera constitute the most important differentiating factors between PD and controls, we performed random forests classification in MicrobiomeAnalyst, as described previously (37). To assess the correlation of disease duration and abundance of genera, and the influence of UPDRS-III and levodopa equivalent dose (LEDD), we used the processed, normalized and scaled abundances from MicrobiomeAnalyst in IBM SPSS statistics version 24.0 (IBM). A partial correlation was performed with a significance level of 0.05, two-tailored using zero-order correlations with UPDRS-III and LEDD as covariates.

As microbiome data are compositional, analyzing differences between groups using classical statistical testing may lead to false assumptions of differences in microbiome composition, a factor that may be large in some datasets, and small in others (40). To further substantiate our findings, we used the pattern search feature of MicrobiomeAnalyst. We specified either group (ctrl vs. PD) or disease stage (controls vs. H&Y1 vs. H&Y2 vs. H&Y3 vs. H&Y4) as a feature. Sparse Correlations for Compositional data (SparCC) (40) was used as distance measure of taxa between features. As SparCC assumes a sparse network and uses log-ratio transformed data and performs iterations to identify correlations that are distinct from correlations resulting from network changes within a compositional dataset, the results of this pattern search approach take the compositional character of our data into account.

Finally, to analyze confounders such as by coffee consumption, NMS, and in particular constipation, we performed propensity score matching using the balanced, parallel, variable ratio (1:*n*) nearest-neighbor approach (41) in SPPS® and R (V 2.14.2; The R Foundation for Statistical Computing, Vienna, Austria). Matching was performed to control for the covariates total NMS score, NMS constipation item, coffe consumption, and Wexner Constipation Score. Matched groups were analyzed for differences in microbiota composition in MicrobiomeAnalyst, analog to unmatched groups.

## Results

### Demographics and Clinical Data

**Figure 1:**
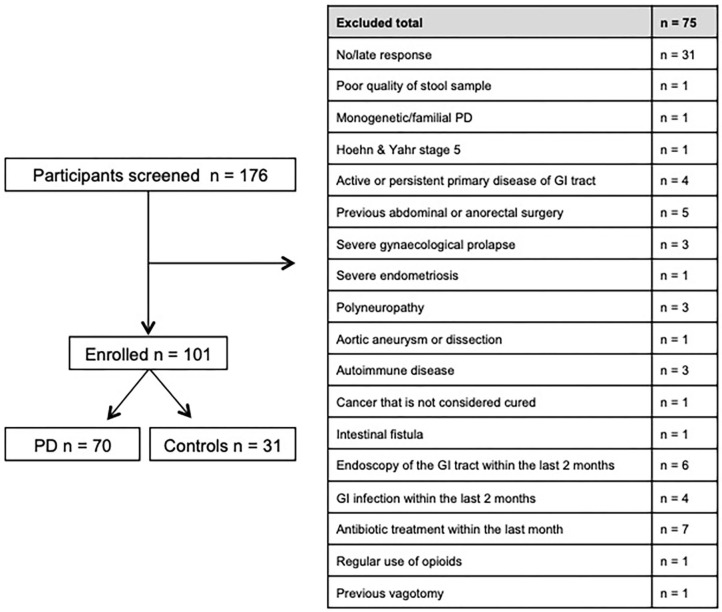
Recruitment process and main exclusion criteria.

Between November 2016 and June 2018 176 subjects were recruited. Of these, 101 (70 PD, 31 controls) fulfilled the inclusion criteria (Figure 1). There was no difference in gender distribution between groups (patients 45.7% females; controls 45.2% females). In addition, mean age (PD: 65.3 yrs ± 10.2; controls: 64.3 yrs ± 8.9) did not differ between groups. Mean disease duration of the PD group was 7.4 years (±5.7 yrs) and mean H&Y was 2.2 (±1.0). Mean LEDD was 660.9 mg (±563.9 mg) per day (Table 1). As expected, overall NMS score was significantly higher in PD patients than in controls (7.1 ± 4.8 vs. 2.7 ± 2.2; *p* < 0.001), as was the frequency of constipation reported in the NMS questionnaire (32.9 vs. 6.5%, *p* = 0.005). Also, Wexner Constipation Scores were significantly higher in PD patients than in controls (4.2 ± 3.8 vs. 2.3 ± 1.9; *p* = 0.034). Changes in frequency of incomplete bowel emptying, as reported in the NMS questionnaire was altered, however bordered significance, only (22.9 vs. 6.5%; *p* = 0.053). Half of PD patients and 0% of controls were screened positive for iRBD (Table 1). Patients and controls did not differ in smoking, use of alcohol, intake of prebiotic and probiotic supplements as well as consumption of probiotic yogurt, regular yogurt and natural food sources occurring in Western diet, containing a relevant amount of the prebiotics inulin and oligofructose (42). Both groups showed no difference in self-reported consumption of additional salt during meals. However, PD patients showed a significantly lower coffee intake compared to controls (*p* < 0.001). Moreover, PD patients and controls were similarly active regarding easy to moderate physical exercise. A detailed summary of the assessed covariates is provided in Supplementary Table 2. As expected, control subjects were more involved in demanding physical activity than PD patients. Except for dopaminergic medication, there was no difference between groups in medication intake (Tables 2, 3).

**Table 1:** Demographics, lifestyle factors and comorbidity PD vs. controls.

	**PD**	**Control**	***P*-value**
*N*	70	31	
Female subjects	45.7%	45.2%	1.000^a^
Mean age	65.3 ± 10.2	64.3 ± 8.9	0.651^b^
Mean weight [kg]	76.4 ± 15.6	77.8 ± 14.1	0.670^b^
Mean height [cm]	171.1 ± 10.5	170.1 ± 7.7	0.700^c^
Mean H&Y stage	2.2		
Mean disease duration	7.4 years		
Mean LEDD	660.88 mg		
**Severity of NMS**			
mean NMS Score	7.1 ± 4.8	2.7 ± 2.2	<0.001^c^
NMS item “constipation”	32.9%	6.5%	0.005^a^
NMS item “bowel emptying incomplete”	22.9%	6.7%	0.053^a^
Degree of constipation symptoms (Wexner)	4.2 ± 3.8	2.3 ± 1.9	0.034^c^
**Lifestyle factors**			
Smoking	1.4%	6.5%	0.222^a^
Coffee consumption >2 cups per day	18.6%	58.1%	<0.001^a^
Alcohol consumption ≥ twice a week	45.7%	56.7%	0.385^a^
Probiotic supplement consumption	4.3%	3.2%	>0.05^d^

a Fisher's exact test;

b t-test;

c Mann-Whitney U-test;

d *Chi-square test with Bonferroni correction*.

**Table 2:** Antiparkinsonian medication intake in PD vs. controls.

**Antiparkinsonian medication**	**PD**	**Controls**	***P*-value**
Levodopa	71.4%	0.0%	<0.001^a^
COMT inhibitor	15.7%	0.0%	0.017^a^
Dopamine agonist	78.6%	0.0%	<0.001^a^
MAO-B inhibitor	62.9%	0.0%	<0.001^a^
Amantadine	18.6%	0.0%	0.008^a^
Anticholinergic	0.0%	3.2%	0.304^a^

a *Fisher's exact test (COMT, Catechyl-O-methly transferase; MAO-B, Monoaminooxidase-B)*.

**Table 3:** Antiparkinsonian medication intake in different H&Y stages vs. controls.

**Antiparkinsonian medication**	**H&Y 1**	**H&Y 2**	**H&Y 3**	**H&Y 4**	**Controls**	***P*-value**
L-Dopa	26.7%^a^	78.8%^ab^	83.3%^ab^	100%^ab^	0.0%^c^	<0.001^1^
COMT inhibitor	0.0%^a^	12.1%^a^	8.3%^a^	60.0%^b^	0.0%^a^	<0.001^1^
Dopamine agonist	86.7%^a^	78.8%^a^	75.0%^a^	70.0%^a^	0.0%^b^	<0.001^1^
MAO-B inhibitor	73.3%^a^	69.7%^a^	50.0%^a^	40.0%^a^	0.0%^b^	<0.001^1^
Amantadine	6.7%^a^	18.2%^a^	25.0%^ab^	30.0%^ab^	0.0%^c^	0.010^1^
Anticholinergic	0.0%	0.0%	0.0%	0.0%	3.2%	0.670^1^

1 *Fisher's exact test with Bonferroni correction. Letters a, b, and c indicate groups that do not differ on a 0.05 significance level*.

There was no difference between controls and different H&Y stages regarding gender distribution, weight and height. Patients in H&Y stage 1 were younger than patients in more advanced disease stages and controls. Neither frequency of constipation reported in the NMS questionnaire nor absolute Wexner Constipation Score showed overall differences between H&Y stages. Also, coffee consumption did not differ between different disease stages. See Supplementary Table 3 for details on assessed covariates regarding overall group differences between controls and different H&Y stages.

### Diversity Measures of the Microbiota

There were no differences in Shannon (*p*: 0.10879; *t*: 1.6207), and Chao1 (*p*: 0.83138; *t*: −0.21396) alpha-diversity indices. Simpson index showed a significantly reduced alpha diversity (*p*: 0.0080764; *t*: 2.7035) between controls and PD patients (Supplementary Figure 1). There were no differences in alpha-diversity between controls and H&Y stages 1–4 (data not shown). A difference in beta-diversity (Bray Curtis index) was observed between controls and PD (PERMANOVA: F:1.7844; *p* < 0.047; Supplementary Figure 1), and when comparing H&Y stages 1–4 and healthy controls (PERMANOVA: F:1.5651; *p* < 0.008; Supplementary Figure 2).

### Altered Gut Microbiota in PD Patients on Different Taxonomic Levels

**Figure 2:**
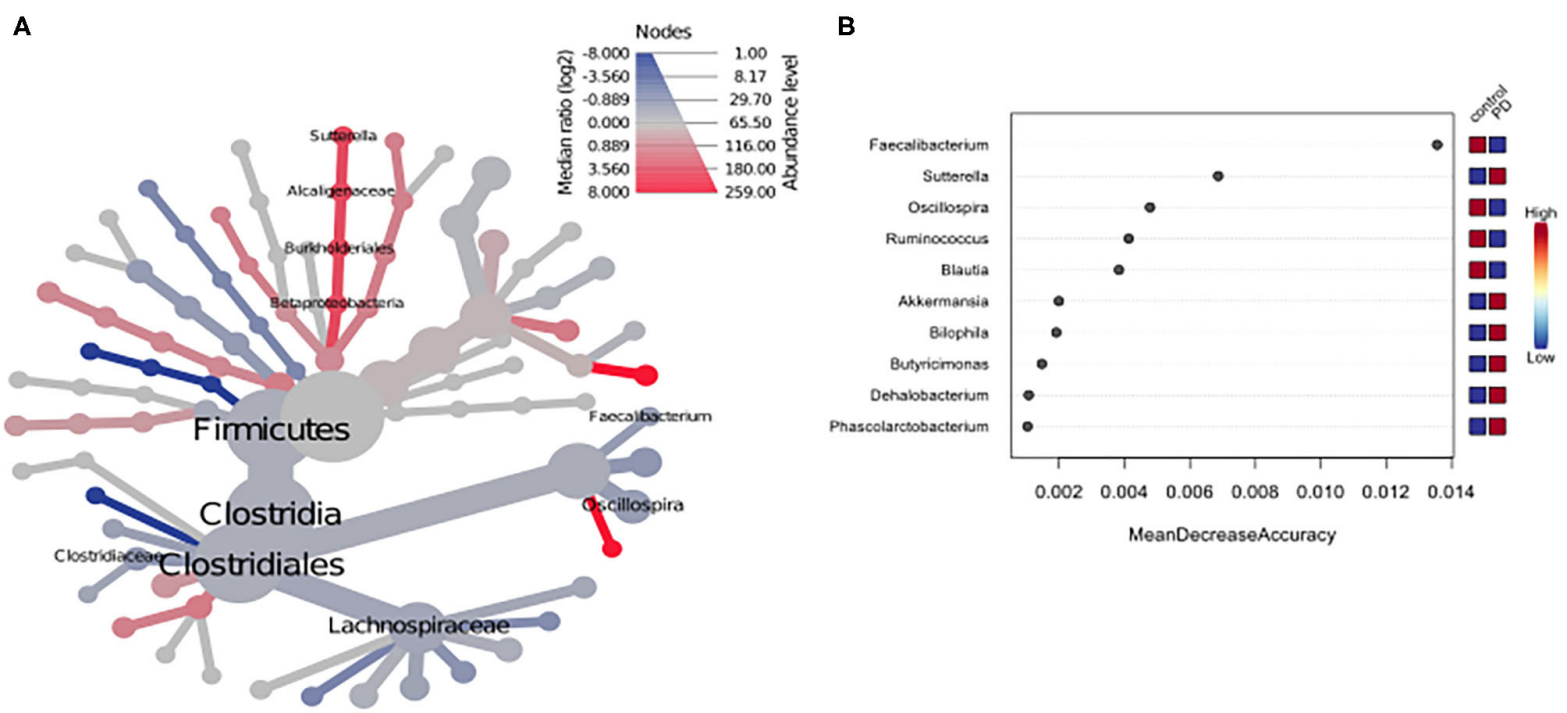
**(A)** Heat tree analysis depicting alterations in microbiota composition between PD and controls. Significantly altered taxa are displayed by name at the corresponding node. Nodes indicate the hierarchical structure of taxa. A red branch indicates an increase in PD compared to controls, while a blue branch indicates a decrease. **(B)** Unsupervised random forests classification using PD and controls as classes and abundances as classifiers along a decision tree. Y-axis: 10 most important classifying variables, z-axis: mean decrease accuracy (MDA) is a measure of loss of accuracy if the classifier on the y-axis is removed from the classification. Mini heatmap (Blue or red squares) on the right indicate the abundance in the groups (red indicates high abundance, blue low abundance).

**Figure 3:**
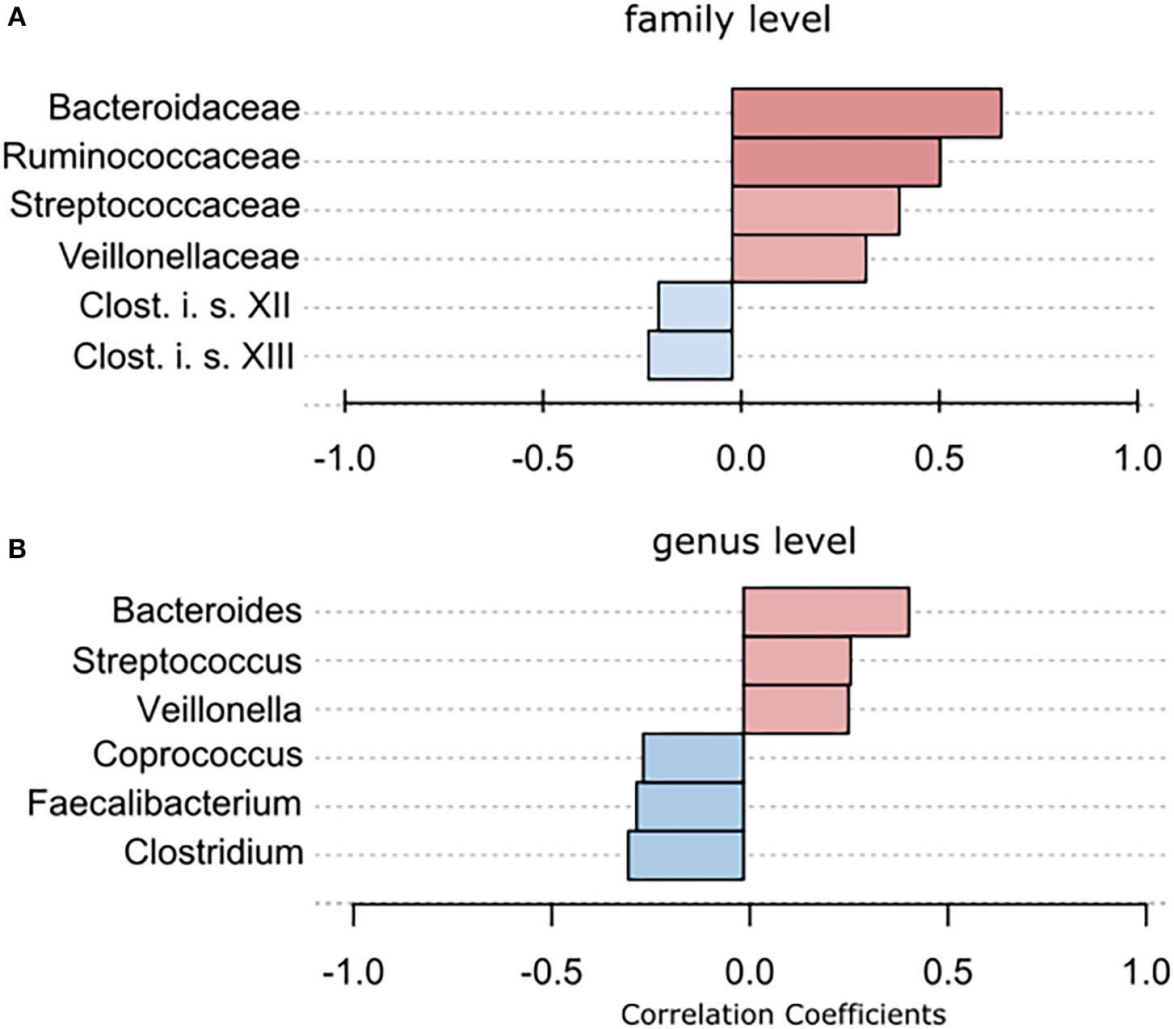
To further strengthen the described alterations via a compositionally aware method, we used pattern search in Microbiomeanalyst with SparCC as distance measure to identify correlations of individual taxa with groups. **(A)** Bars indicate the value for the correlation coefficient of a significantly correlated taxon with PD on **(A)** family level and **(B)** genus level. Correlation coefficients are depicted as positive (red) or negative correlations (blue). In example, on family level, a correlation coefficient of 0,679 between Bacteriellaceae and PD was observed, this correlation was significant (*p* = 0.0099).

Using classical group comparison, PD patients displayed several alterations of individual taxa (summarized in Supplementary Figure 3). Using a Wilcoxon-signed rank test (using heat tree analysis in MicrobiomeAnalyst), we solely observed a decreased abundance of Firmicutes on phylum level (*p* < 0.042). We observed an altered microbiota composition within the class Clostridia (*p* = 0.0301), order Clostridiales (*p* = 0.0301), family Lachnospiraceae (*p* = 0.0079), and family Clostridiaceae (*p* = 0.0491). Within the family of Ruminococcaeceae (*p* = 0.161), the genus *Ruminococcus* was not altered significantly (*p* = 0.067), however the genus *Faecalibacterium* (*p* = 0.017) was significantly altered, as well as *Oscillospira* (*p* = 0.0421). Within the class Betaproteaobacteria (*p* = 0.0038), order Burkholderiales (*p* = 0.0038), family Alcaligenaceae (*p* = 0.0038), the genus *Sutterella* (*p* = 0.0038) was significantly altered in PD (Figure 2A). Random forests classification (OOB error = 0.0337, classification error for PD = 0.0714) further substantiated a role of *Faecalibacterium, Sutterella, Oscillospira, Ruminococcus*, and *Blautia* as the 5 most important differentiating factors, separating PD microbiota from controls (Figure 2B). Using pattern search in MicrobiomeAnalyst with SparCC as distance measure, we did not observe any correlation of a certain phylum with the feature group (controls vs. PD). Using this method, on class level, only Gammaproteobacteria were significantly correlated with PD (*r* = 0.5355; *p* = 0.0297; Supplementary Figure 4). On family level, Bacteroidaceae (*r* = 0.6794, *p* = 0.0099), Ruminococcaceae (*r* = 0.5249, *p* = 0.099), Streptococcaceae (*r* = 0.4216, *p* = 0.0099), and Veillonellaceae (*r* = 0.3377, *p* = 0.0297), showed positive correlations with groups, whereas Clostridiales incerte sedis XII (*r* = −0.2625, *p* = 0.0396) and XIII (*r* = −0.2113, *p* = 0.0495) were negatively correlated with groups (Figure 3A). On genus level, *Clostridium* (*r* = −0.291, *p* = 0.0198), *Bacteroides* (*r* = 0.4652, *p* = 0.0297), *Streptococcus* (*r* = 0.2704, *p* = 0.0297), *Veillonella* (*r* = 0.2646, *p* = 0.0297), *Faecalibacterium* (*r* = −0.27, *p* = 0.0396), and *Coprococcus* (*r* = −0.2532, *p* = 0.0495) (Figure 3B) were correlated with PD. In summary, using several statistical methods to address alterations in PD, *Faecalibacterium* was the most consistently altered taxon across tests.

### The Influence of Disease Stage on the Abundance of Different Genera

Using classical non-parametric group comparison, there were significant differences in the abundance of several bacteria on different taxonomic levels between controls and individual H&Y stages (Supplementary Table 4). Pattern search using disease stage as factor and SparCC as distance measure revealed that on class level Gammaproteobacteria was correlated with disease stage. On genus level, in addition to *Faecalibacterium*, also *Bacteroides, Clostridium, Phascolarctobacterium, Coprococcus, Odoribacter* were correlated with disease stage. On family level, Bacteroidaceae and Turicibacteraceae correlated with disease stage (Table 4).

**Table 4:** Significant correlations of microbiota with disease stage.

	**Correlation**	***P*-value**	**c, 1, 2, 3, 4**	
**Class**				
Gammaproteobacteria	0.541	0.0198	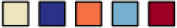	
**Family**				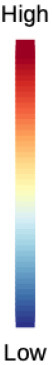
Bacteroidaceae	0.3616	0.0495	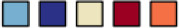	
Turicibacteraceae	0.2205	0.0495	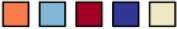	
**Genus**				
Bacteroides	0.4491	0.0198	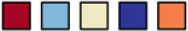	
Faecalibacterium	−0.3854	0.0198	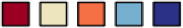	
Clostridium	−0.297	0.0297	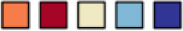	
Phascolarctobacterium	0.2442	0.0297	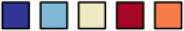	
Coprococcus	−0.2965	0.0495	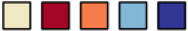	
Odoribacter	−0.2441	0.0495	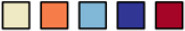	

*Correlations coefficients, and p-values are given. The third column shows a mini heatmap (37). Colors indicate high (red spectrum) or low abundances (blue spectrum) of the taxon in the corresponding disease stage. c, ctrls 1,2,3,4 represent corresponding H&Y stages. In example, on genus level, Faecalibacterium was negatively correlated with disease stage. Thus, mini heatmap shows high abundances in c, and low abundances in H&Y stages 3 and 4*.

### The Influence of Disease Duration on the Abundance of Different Genera Depicts an Important Contribution of Dopaminergic Medication

Disease duration correlated with *Faecalibaterium* (*p* < 0.043, *r* = −0.244), *Parabacteroides* (*p* < 0.016, *r* = 0.29), and *Turicibacter* (*p* < 0.041, *r* = 0.247; Table 5A). Since disease duration leads to increasing doses in dopaminergic medication and is associated with increased motor impairment, we performed a partial correlation analysis to assess the effect of the LEDD (Table 5B), UPDRS III (Table 5C) as well as LEDD and UPRDS III (Table 5D) on the correlation of disease duration with genera. LEDD, UPDRS III, and LEDD + UPDRS III differently impacted correlation. While the significant positive correlation of *Parabacteroides* and *Turicibacter* with disease duration was maintained after controlling for LEDD, UPDRS III and the combination of both, correcting for LEDD led to a loss of significance for *Faecalibacterium*. Correcting for the covariate UPRDS III did not affect correlations. For *Sutterella*, correcting for the LEDD strengthened the correlation, bordering significance. Thus, dopaminergic medication, but not motor impairment may influence microbiome composition upon disease progression.

**Table 5:** Partial correlation between (A) genera and disease duration (in years), assessing the effects of covariates (B) levodopa equivalent dose (LEDD), (C) motor impairment (UPDRS III), and (D) the combination of LEDD and UPDRS III.

**Disease duration**								
**Covariates**	**(A)**		**(B) LED**		**(C) UPDRS-III**		**(D) LED** **+** **UPDRS-III**	
	***r***	***p*-value**	***r***	***p*-value**	***r***	***p*-value**	***r***	***p*-value**
Akkermansia	0.077	0.530	0.050	0.687	0.074	0.546	0.050	0.690
Blautia	−0.100	0.414	0.020	0.871	−0.120	0.327	0.021	0.869
Clostridium	−0.193	0.112	−0.151	0.220	−0.187	0.125	−0.150	0.225
Faecalibacterium	**−0.244**	**0.043**	−0.159	0.194	**−0.265**	**0.028**	−0.160	0.197
Lachnospira	−0.132	0.279	−0.053	0.665	−0.130	0.286	−0.053	0.670
Oscillospira	−0.074	0.544	0.103	0.405	−0.060	0.624	0.107	0.390
Parabacteroides	**0.290**	**0.016**	**0.245**	**0.044**	**0.279**	**0.020**	**0.247**	**0.044**
Prevotella	0.089	0.469	0.001	0.991	0.095	0.437	0.002	0.986
Roseburia	−0.235	*0.052*	−0.138	0.263	−0.232	*0.055*	−0.137	0.268
Ruminococcus	0.121	0.322	0.110	0.370	0.101	0.409	0.111	0.370
Sutterella	–*0.213*	*0.079*	–*0.231*	*0.058*	–*0.207*	*0.088*	–*0.231*	*0.060*
Turicibacter	**0.247**	**0.041**	**0.296**	**0.014**	**0.248**	**0.040**	**0.296**	**0.015**

*r, pearson's correlation coefficient. Bold letters: indicates significant changes; italic letters: has no meaning*.

### The Influence of Constipation and Coffee Consumption

**Figure 4:**
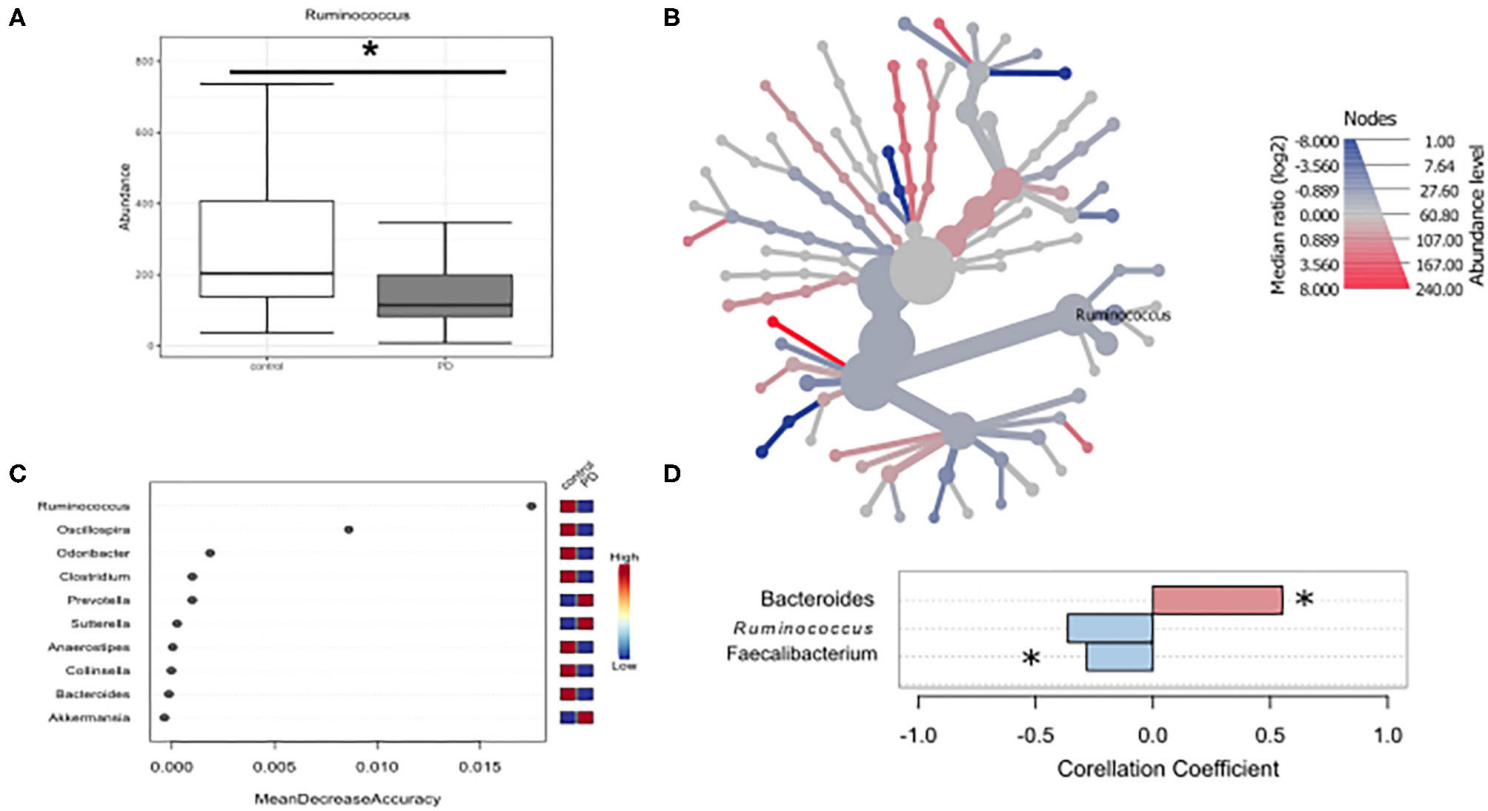
Differences in microbiota after matching for obstipation and coffee consumption. **(A,B)** *Ruminococcus* remains the only reduced genus after propensity score matching using non-parametric statistics. **(C)** Random forests classification supports this finding. **(D)** To further strengthen these results, we used pattern search in Microbiomeanalyst with SparCC as distance measure. With this approach, Ruminococcus (*r* = 0.3926) is not correlated significantly with PD, while Bacteroides and Faecalibacterium are. * indicates *p* < 0.05.

Given that microbiota composition is influenced by a multitude of factors other than disease duration and motor impairment, this study was designed to include participants based on stringent inclusion criteria, already controlling for a large variety of covariates upon enrollment. Yet, PD associated covariates cannot be controlled for a priori. To investigate the influence of these covariates on microbiota composition, we preformed propensity score matching of PD patients and controls for those covariates that showed significant differences between controls and PD, NMS score, Wexner Constipation Score and coffee consumption. Matching led to two study groups not differing concerning these covariates (PD *n* = 28; controls *n* = 19; Table 6). Also, matched groups did not differ in demographics and clinical data apart from RBDSQ ratings and dopaminergic medication (see Supplementary Table 5). After matching, one bacterial genus was significantly altered using Mann-Whitney *U* (*p* < 0.05, Figure 4A), and Wilcoxon signed rank test (*p* = 0.0187, Figure 4B): *Ruminococcus* was reduced in PD patients compared to controls, and could was identified as the most important classifier (Figure 4C). Using groups (controls vs. PD) as a factor in pattern search, and SparCC as distance measure, we observed a correlation of PD with *Bacteroides* (*r* = 0.4924, *p* = 0.0396), and *Faecalibacterium* (*r* = −0.2827, *p* = 0.0099), only. *Ruminococcus* (*r* = −0.3926, *p* = 0.1386) did not show a significant correlation with PD (Figure 4D). Thus, PS matching lead to a loss of significance as well as correlation of most taxa. This indicates, that PD associated covariates have a profound impact on microbiome composition, likely contributing to the observed alterations in microbiota in PD.

**Table 6:** Demographics, lifestyle factors and comorbidity of PD vs. controls after propensity score matching for NMS score, NMS item constipation, Wexner constipation score, and coffee consumption.

	**Patients**	**Controls**	***P*-value**
*N*	28	19	
Female subjects	50.0%	52.6%	1.000^a^
Mean age	62.6 ± 12.1	64.7 ± 10.7	0.556^b^
Mean weight [kg]	76.3 ± 17.7	78.9 ± 15.6	0.610^b^
Mean height [cm]	170.9 ± 11.6	169.7 ± 7.6	0.683^b^
**Lifestyle factors**			
Smoking	3.7%	10.5%	0.561^a^
Coffee consumption >2 cups per day	32.1%	52.6%	0.228^a^
Alcohol consumption ≥ twice a week	53.6%	52.6%	1.000^a^
Probiotic supplement consumption	7.1%	0.0%	>0.05^d^
**Comorbidities**			
Mean NMS score	3.6 ± 3.0	3.4 ± 2.3	0.879^c^
NMS item “constipation”	10.7%	10.6%	1.000^a^
Degree of constipation symptoms (Wexner score)	2.5 ± 2.7	2.4 ± 2.3	0.996^c^

a Fisher's exact test;

b t-test;

c Mann-Whitney U-test;

d *Chi-square test with Bonferroni correction*.

## Discussion

We provide further evidence for altered abundances within the gut microbiota in PD. We demonstrate that disease stage and duration impact microbiota composition. PD associated NMS profoundly impact the microbiota as evidenced by the very limited residual differences in microbiota composition after controlling for these covariates. Overall, *Faecalibacterium* was the most consistently decreased taxon in PD across all tests used in this study. Our results especially highlight the pivotal influence of constipation and coffee consumption on microbiota composition. Prior to matching, groups also differed in the practice of demanding physical activity known to impact the gut microbiota (43). Yet, matching also ameliorated this covariate.

There is growing evidence of the interaction of gut microbiota and dopaminergic medication (19, 20). Also, differences in the gut microbiota as a function of anticholinergics have been described before (19). Thus, one major limitation of comparing PD patients to controls is the fact that the effect of dopaminergic medication cannot be controlled for. In this regard, two studies have investigated the microbiome composition in drug naïve PD, so far. Both studies revealed altered gut microbiota (15, 44). In our cohort, there was a difference in the intake of dopaminergic medication including iCOMT and Levodopa (Tables 2, 3). Alterations of iCOMT (19) and Levodopa (20) via the microbiota have recently been studied, indicating that in particular Enterococcus faecalis metabolizes Levodopa. Thus, Levodopa intake may result in a relative overgrowth of this taxon. We show that LEDD is an important covariate influencing correlation of abundances between genera and disease duration, however no difference in the abundance of Enterococcus faecalis was observed. Since Levodopa metabolism takes place in the upper small intestine, the reliability of stool samples to address the interplay of medication on microbiome composition may be further limited. This is of particular importance since there are intraindividual differences between mucosal and fecal compositions of microbiota (45), also in PD (46). Thus, a major limitation of this study is the lack of analysis of mucosal microbiota composition via gastrointestinal biopsies. As observed alterations in stool microbiome composition may not reflect mucosal microbiome composition sufficiently, results on the effect of taxa on gut i.e., medication metabolisms, and gut inflammation, appearing at distinct sites within the gut must be interpreted with caution. In this regard, Keshavarzian et al. observed an increase of anti-inflammatory butyrate-producing bacteria in fecal samples compared to mucosal samples (46). Thus, it is important to further understand which role adherent mucosal compared to fecal microbial communities in the gastrointestinal tract play and how they interact.

### Methodological Considerations

For this study, the criteria of the human microbiome project from 2013 were followed. Also, this study was designed to be in analogy with the pilot study by Scheperjans et al., well knowing that for such a highly active field of research, criteria have already advanced. The field of microbiome research has been made possible by recent advances in sequencing technologies and undergoes constant developments and advances. In this context, decreasing costs for metagenomic shotgun sequencing and the increasing transportability of sequencing technology (e.g., nanopore sequencing®) may enable large-scale, high quality microbiota research overcoming the heterogeneity of current sequencing approaches as well as the remaining shortcomings of 16s RNA sequencing, especially on species level (38). This is currently the most common method used and relies on sequencing of variable regions within bacterial 16s rRNA. Since several variable regions of the 16s rRNA can be used for taxonomic assignment, differences may occur due to the variable regions selected. Regarding microbiome studies in PD, 13 studies used high throughput 16s rRNA gene sequencing. Of these, six studies used the V3–V4 region (15, 47–51), one relied on V3–V5 (52), one on V1–V2 (53), four on V4 only (16, 19, 46, 54), and one used the V1–V3 (29) region for taxonomic classification. Two studies used quantitative RT-PCR (55, 56), one used metagenomic shotgun sequencing (44). In this regard, results using Illumina MISeq sequencing V3–V4 region are probably the most comparable to the results reported in this study. However, the six studies using the V3–V4 region for sequencing mentioned above varied in stool collection methods, e.g., use of stabilizing solution and storage temperature. Furthermore, no information is provided on how participants were instructed to gain the sample, e.g., use of a stool collection aid to prevent contamination as used in this study. Delineating the influence of the collection method and the rRNA sequencing approach on microbiome composition in PD would give important insights for planning of future trials on microbiota composition in PD. Despite these methodological differences, two recent reviews suggest an increasing comparability of microbiota composition across studies (14, 17). In addition to sample collection and sequencing, in particular statistical methods used to analyze microbiome data have a profound impact. Since microbiome data are compositional (57), the most reliable method used in our study is probably pattern search using SparCC to assess differences in taxa between PD and controls or between disease stages (40).

### Alterations of Diversity Indices Are Not a Major Feature in PD

Diversity indices are core features of microbiome analysis, addressing global features of microbiota composition. Overall, alpha-diversity as a measure for number of bacterial taxa in individual stool samples did not clearly differ in PD vs. controls, a common finding across most microbiome studies in PD apart from the studies by Kesharvazian et al. (46), Baricella et al. (15), and Qian et al. (50) reporting decreased alpha-diversity, and one single study by Petrov et al. reporting increased alpha diversity (47). Therefore, a simple change in microbiota diversity does not appear to be a key feature of PD. Beta diversity, as a measure of similarity of microbiota composition within a group was not changed between controls and PD. In total, 13 studies have reported differences in ß-diversity before (15, 16, 19, 29, 44, 46–48, 50–54). In this study a significant, yet small increased beta-diversity was observed between controls and PD. Most of this effect appears to be attributable to H&Y stages 1–2 vs. controls and H&Y stage 4 (Supplementary Figure 2). This indicates that at these stages, the microbiota composition amongst PD patients is more dissimilar than in controls, which points toward a subordinated role of a specific composition of gut bacteria in PD, which would be associated with lower beta-diversities, but is in favor of external factors influencing microbiota composition in PD. A variety of PD related factors that differ across disease stages may account for these differences (Supplementary Table 3), yet analyzing their individual contribution to dissimilarity by i.e., factor analysis or MANCOVA would deliver robust results only with a higher number of data points.

### Increasing Comparability Across Microbiota Studies

Several findings in this study are in line with previously published data. We observed a decreased abundance for the genus *Ruminococcus*, which remained significant after propensity score matching, making it one of the consistently altered parameter across all tests performed in this study. Reduced abundances of the genus *Faecalibacterium*, belonging to the family Clostridiaceae have been reported in four studies: one North American study (19), one Russian (47), and two Chinese studies (52, 54), indicating a regionally independent influence in PD. In our study this taxon was consistently reduced in PD across all tests used in this study. We observed a significant correlation with disease duration and a prominent influence of H&Y stage 4, suggesting a role of covariates appearing in late PD. Thus, this study adds evidence to alterations in these taxa, particularly in late PD.

Even though we observed several taxa to be altered within the phylum Bacteroidetes, and Proteobacteria, we did not observe changes on phylum level in PD, apart from differences in Firmicutes. Taken together, certain bacterial families are consistently altered in PD across different studies. In particular decreased abundances in the phylum Firmicutes, class Clostridia, order Clostridiales are repeatedly reported. On the contrary, Proteobacteria, namely the family of Enterobacteriaceae and Sutterellaceae appear to be increased in PD. Our data indicate that disease duration and disease stage significantly influence these alterations. Amongst the variety of covariates influencing the microbiota, PD associated alterations cannot be controlled for a priori. To assess the contribution of these covariates we chose to perform propensity score matching, controlling for total NMS score, NMS constipation item, Wexner Constipation Score and coffee consumption, since these were altered compared to controls in our cohort. Since only *Bacteroides, Faecalibacterium*, and probably *Ruminococcus* remained reduced after matching, these taxa may be altered in PD independent of coffee consumption and obstipation.

### Clinical Relevance and Future Perspectives

In summary, changes in microbiota composition occur in PD. Even though studies vary in power, inclusion criteria, methodology and regionality, several taxa are similarly altered across studies. Yet, a consistent picture is still missing. We focused our study on the influence of covariates on microbiome composition in PD, and show that disease duration and disease stage influence microbiota. The effect of disease stage and duration is noteworthy, since future studies should focus on early disease stages (H&Y <2) to obtain more robust data on microbiota alterations that may be susceptible to modification strategies. Here, follow-up results on microbiota in iRBD converting to early PD (16) would be of particular interest. Pipelines for analyzing longitudinal changes of microbiota composition are under development (58), but their interpretation still poses problems. In addition, we describe a crucial role of the PD associated covariates constipation and coffee consumption, as well as dopaminergic medication. Even though these results are not novel, they highlight the need to take these covariates into account when designing future studies on microbiome composition in PD. While coffee consumption and constipation may be controlled for by inclusion criteria and subsequent matching, the effect of dopaminergic medication can only be addressed by including drug naïve PD cohorts into microbiome studies. Even more so, mechanistic information to determine the effective role of the microbiota in PD is necessary to identify putative treatment options under discussion (59). This is one major limitation of microbiome studies, which are solely based on 16s rRNA sequencing data. Recent studies have started to overcome these limitations by combining classical 16s RNA microbiome analysis with PCR analysis from stool for key enzymes of metabolic pathways and metabolome analysis of serum levels to assess functional consequences (60). Yet, the most stringent proof for a functional role of microbiota in PD would be the functional prediction of shotgun sequencing data derived from mucosal biopsies in drug naïve PD and parallel stool and serum metabolome analysis.

## Data Availability Statement

The raw data supporting the conclusions of this article will be made available by the authors, without undue reservation, to any qualified researcher.

## Ethics Statement

The study was approved by the local ethics commission (No. 284_16 B), and all participants gave written informed consent.

## Author Contributions

AC-G and FM: participant recruitment, handling of stool samples, DNA extraction, statistical analysis, writing the manuscript, and study concept. HM: handling of stool samples and DNA extraction. AM: statistical analysis, propensity score matching, and revision of manuscript. SW: sequencing and bioinformatical processing, revision of manuscript, and study concept. JW: participant recruitment, revision of manuscript, and study concept AC-G: performed the present work in fulfillment of the requirements for obtaining the degree Dr. med. All authors: contributed to the article and approved the submitted version.

## Conflict of Interest

The authors declare that the research was conducted in the absence of any commercial or financial relationships that could be construed as a potential conflict of interest.
