# Supplementary material for: Changes in Gastrointestinal Microbiome Composition in PD: A Pivotal Role of Covariates
Source: Front Neurol. 2020 Sep 23;11:1041. doi: 10.3389/fneur.2020.01041 (PMC7538808; doi:10.3389/fneur.2020.01041)
Supplement: Supplementary file 1 [file Data_Sheet_1.PDF]

supplementary figure 1: diversity indices PD vs controls

alpha diversity

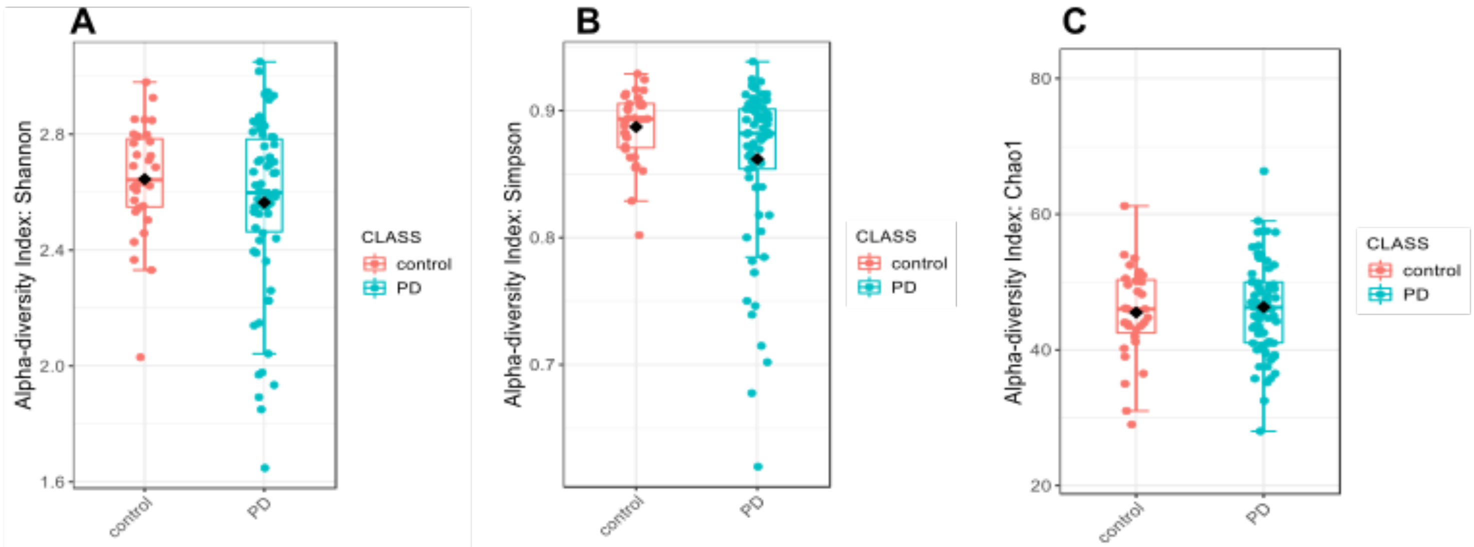

p-value: 0.10879; t: 1.6207

p-value: 0.0080764; t: 2.7035

p-value: 0.83138; t: -0.21396

beta diversity

[PERMANOVA] F-value: 1.7844; R-squared: 0.017705; p-value < 0.047

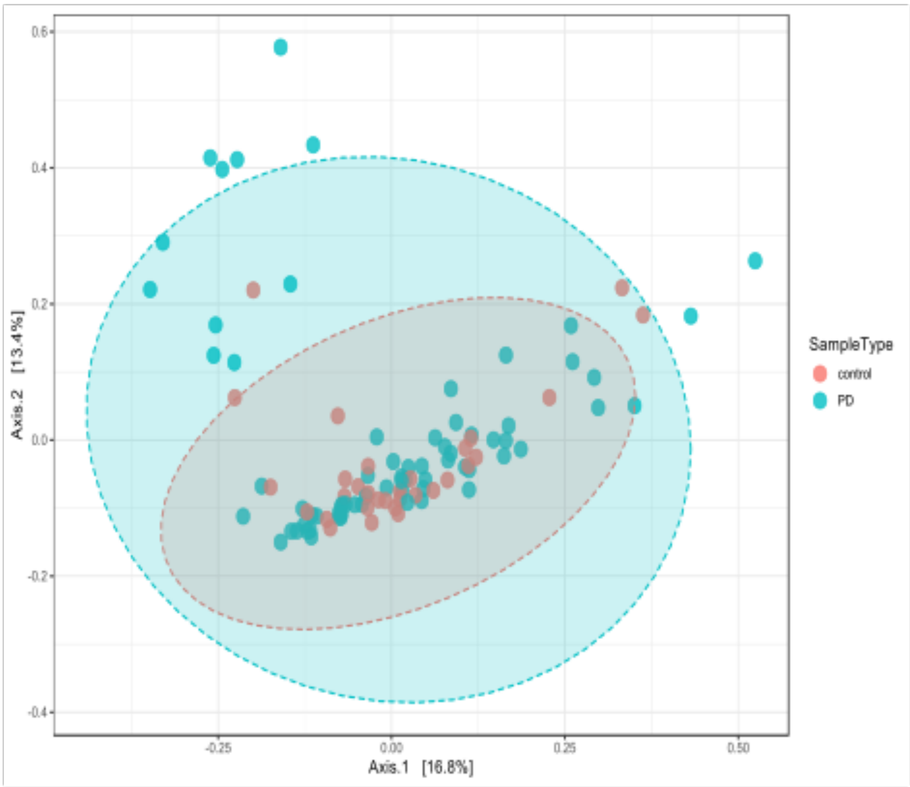

supplementary figure 2 beta diversity across disease stages

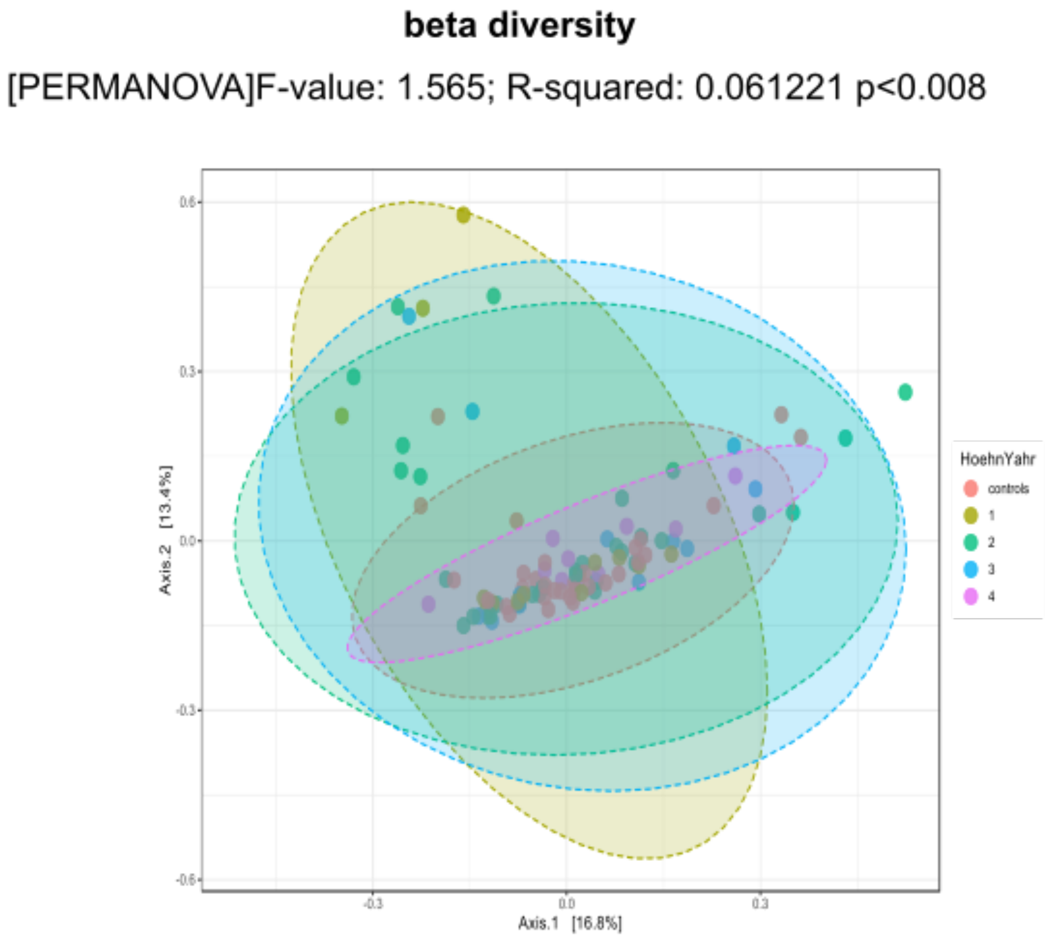

supplementary figure 3: altered abundances across Taxa in PD vs controls

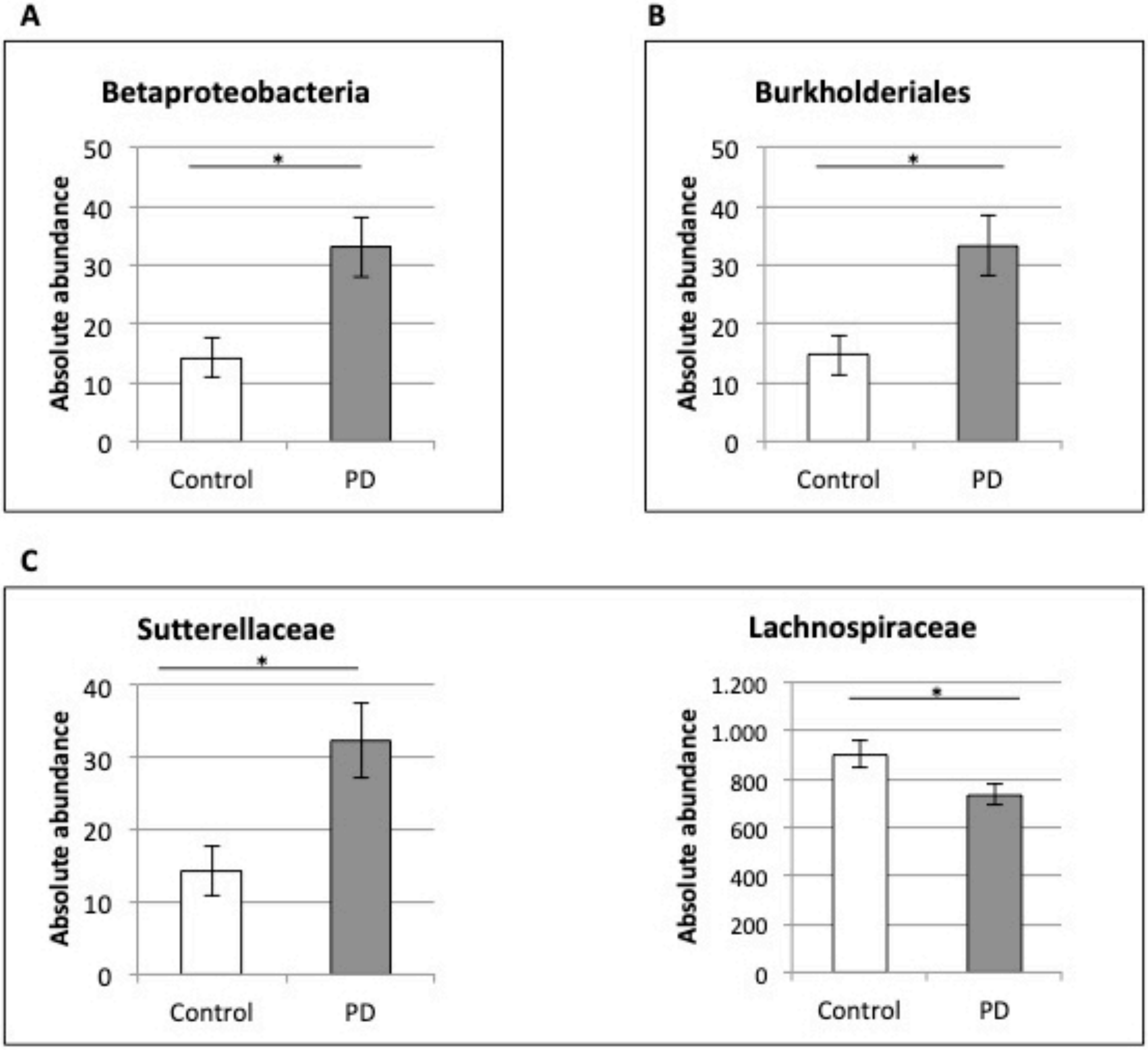

D

Genus	Controls	PD	P-value
Faecalibacterium	263.16 ± 26.80	204.01 ± 19.13	0.028
Fusicatenibacter	79.00 ± 12.32	56.57 ± 8.93	0.018
Gemmiger	179.68 ± 23.99	144.43 ± 19.64	0.027
Lachnospiracea incertae sedis	273.26 ± 31.23	196.99 ± 18.08	0.031

Significant differences in bacterial abundancies between controls and PD patients. Bar graphs of PD associated bacterial classes (A), orders (B) and families (C) (* = $p < 0.05$). PD associated bacterial genera (D). Absolute abundances in reads (mean ± standard error).

supplementary figure 4: pattern search depicts differences between PD and controls

A) phylum level:

	Correlation	P-Value
**Proteobacteria**	0.7545	0.2178
**Actinobacteria**	0.247	0.297
**Tenericutes**	-0.1501	0.3069
**Bacteroidetes**	0.8738	0.3168
**Firmicutes**	0.8956	0,396
**Cyanobacteria**	-0.0483	0.8416
**Lentisphaerae**	-0.0297	0.8416
**Verrucomicrobia**	0.0202	0.8713

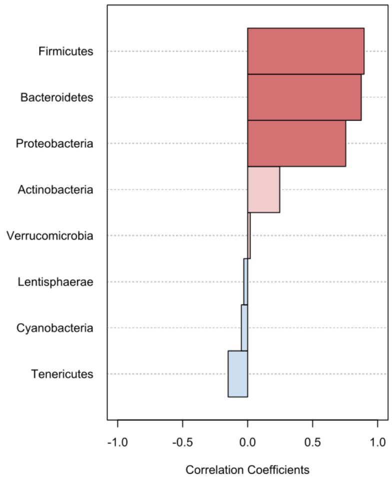

B) class level:

	Correlation	P-Value
**Gammaproteobacteria**	**0.5355**	**0.0297**
**Bacteroidia**	0.8519	0.2574
**Bacilli**	0.173	0.3366
**Clostridia**	0.8699	0.396
**Alphaproteobacteria**	-0.1396	0.4059
**Deltaproteobacteria**	0.0981	0.4851
**Betaproteobacteria**	0.0836	0.5743
**Coriobacteriia**	0.0622	0.703
**Verrucomicrobiae**	0.33	0.7624
**Erysipelotrichi**	-0.03	0.8317
**Lentisphaeria**	0.0101	0.9703
**Actinobacteria**	-0.0109	0.9901

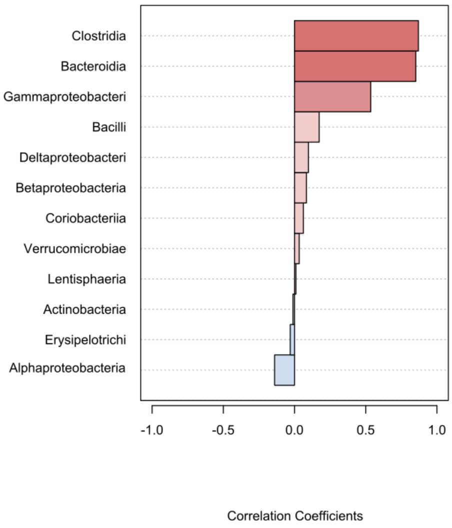

Supplementary figure: Using pattern search with groups (ctrl vs PD) as feature and SparCC as distance measure, no differences between PD and controls on phylum level were observed. On class level, only an increase in gammaproteobacteria was significantly correlated to PD.
