# Supplementary material for: Changes in Gastrointestinal Microbiome Composition in PD: A Pivotal Role of Covariates
Source: Front Neurol. 2020 Sep 23;11:1041. doi: 10.3389/fneur.2020.01041 (PMC7538808; doi:10.3389/fneur.2020.01041)
Supplement: Supplementary file 2 [file Data_Sheet_2.PDF]

Supplementary table 1: Inclusion and exclusion criteria

**Patients**	**Patients and control subjects**	**Control subjects**
	**Inclusion criteria**	
	Written informed consent	
Diagnosis of Parkinson's disease (H&Y stage 1-4)		
	**Exclusion criteria**	
		Any clinical sign of Parkinson's disease
		NMSQuest-Score > 7
		REM sleep behavior disorder (RBDSQ > 4 Punkte)
	Psychosis	
	HIV infection	
	Active or persistent primary disease of gastrointestinal tract: e.g. celiac disease, pernicious anemia, autoimmune gastritis, symptomatic diverticulosis, inflammatory bowel disease, irritable bowel syndrome, strictures, adhesions, varicosis or diverticulum of the esophagus, Meckel's diverticulum	
	Alcohol or drug abuse	
	Active or previous hepatobiliar or pancreatic disease (exception: asymptomatic cholecystolithiasis)	
	Previous abdominal or anorectal surgery (exception: haemorrhoid procedure, 1 year after appendectomy, inguinal hernia repair, gynecological surgery if symptoms resolved)	
	Severe gynaecological prolapse (exception: after repair procedure)	
	Severe endometriosis	
	History of peritonitis, polyneuropathy, polio, spina bifida, paraparesis, symptomatic peripheral arteriosclerosis, intestinal ischemia, aortic aneurysm or dissection, connective tissue disease, autoimmune disease, sarcoidosis outside of lung and skin, active cancer, abdominal, intestinal, or urogenital fistula, severe renal insufficiency (exceptions: Diabetes mellitus, treated thyreoid dysfunction)	
	Within the last 2 months: severe hypokalemia or hyperkalemia,	

	abdominal trauma, gastrointestinal tract infection, food intoxication, major epistaxis, narcosis or analgosedation, endoscopic procedure of the gastrointestinal tract	
	Antibiotic treatment within the last month	
	Use of the following medications: opioids, loperamide, glucocorticoids (oral or parenteral), metoclopramide	

Supplementary table 2: Demographics, lifestyle factors and comorbidity PD vs controls

	Patients	Controls	P-value
**Demographics**			
n	70	31	
Female subjects	45.7%	45.2%	1.000[1]
Mean Age	65.3 ± 10.2	64.3 ± 8.9	0.651[2]
Mean Weight [kg]	76.4 ± 15.6	77.8 ± 14.1	0.670[2]
Mean Height [cm]	171.1 ± 10.5	170.1 ± 7.7	0.700[3]
**Lifestyle factors**			
Smoking	1.4%	6.5%	0.222[1]
Coffee consumption > 2 cups per day	18.6%	58.1%	<0.001[1]
Alcohol consumption ≥ twice a week	45.7%	56.7%	0.385[1]
Probiotic supplement consumption	4.3%	3.2%	>0.05[4]
Prebiotic supplement consumption	1.4%	3.2%	>0.05[4]
Probiotic yogurt consumption ≥ once a week	5.7%	0.0%	>0.05[4]
Regular yogurt consumption ≥ once a week	62.3%	61.3%	>0.05[4]
Kefir or buttermilk consumption ≥ once a week	4.3%	6.5%	>0.05[4]
Banana consumption ≥ once a week	57.1%	67.7%	>0.05[4]
Asparagus consumption ≥ once a week	11.4%	16.1%	>0.05[4]
Chicory consumption ≥ once a week	4.3%	16.1%	<0.05[4]
Dandelion greens consumption ≥ once a week	0.0%	0.0%	>0.05[4]
Garlic consumption ≥ once a week	35.7%	38.7%	>0.05[4]
Artichoke consumption ≥ once a week	0.0%	0.0%	>0.05[4]
Leek consumption ≥ once a week	15.7%	16.1%	>0.05[4]
Onion consumption ≥ once a week	82.9%	87.1%	>0.05[4]
Wholemeal product consumption ≥ once a week	74.3%	77.4%	>0.05[4]
Adding salt to readily cooked dishes "often" or "always"	7.1%	16.1%	>0.05[4]
Easy physical activity [hours/week]	1.9 ± 2.5	1.4 ± 1.7	0.372[3]
Moderate physical activity [hours/week]	1.9 ± 2.4	2.5 ± 2.4	0.102[3]
Demanding physical activity [hours/week]	0.2 ± 1.2	0.9 ± 3.3	0.018[3]
**Comorbidities**			
Mean NMS Score	7.1 ± 4.8	2.7 ± 2.2	<0.001[3]
NMS item "constipation"	32.9%	6.5%	0.005[1]
NMS item "bowel emptying incomplete"	22.9%	6.5%	0.053[1]
Degree of constipation symptoms (Wexner Score)	4.2	2.3	0.034[3]
Degree of depression symptoms (GDS 30)	7.0	4.3	0.037[3]
Probable depression according to GDS 30	17.1%	3.2%	0.061[1]
Degree of REM sleep behaviour disorder symptoms (RBDSQ)	4.7	1.4	<0.001[3]
Probable REM sleep behaviour disorder according to RBDSQ	50.0%	0.0%	<0.001[1]
Restless Legs Syndrome	7.1%	0.0%	0.320[1]
Dementia	1.4%	0.0%	1.000[1]
Diabetes mellitus	1.4%	3.2%	0.522[1]
Thyroid dysfunction (controlled)	10.0%	9.7%	1.000[1]

Cholezystectomy	2.9%	3.2%	1.000[1]
Appendectomy	2.9%	0.0%	1.000[1]
Hysterectomy	0.0%	3.2%	0.307[1]
Gynaecological Prolapse Repair	5.7%	0.0%	0.309[1]
**Medication other than antiparkinsonian drugs**			
Betablocker	17.1%	22.6%	0.584[1]
Selective Serotonine Reuptake Inhibitor	7.1%	0.0%	0.320[1]
Acetylsalicylic Acid	11.4%	9.7%	1.000[1]
Phenprocoumon	1.4%	3.2%	0.522[1]
ACE-inhibitor or ATR-antagonist	28.6%	32.3%	0.814[1]
Diuretic	14.3%	22.6%	0.388[1]
Calcium Channel Blocker	5.7%	16.1%	0.128[1]
Thyroxine	12.9%	9.7%	0.751[1]
Antiepileptic	8.6%	3.2%	0.433[1]
Statin	12.9%	3.2%	0.169[1]
Laxatives	4.3%	0.0%	0.551[1]
Tricyclic Antidepressants	2.9%	0.0%	1.000[1]
Antihistaminics	0.0%	6.5%	0.092[1]
Antiemetics	1.4%	0.0%	1.000[1]
Protone pump inhibitors	11.4%	3.2%	0.268[1]
Hormonal contraception in women	6.2%	7.1%	>0.05[4]
Postmenopausal hormone replacement in women	3.1%	7.1%	0.521[1]

[1]= Fisher's exact test; [2]= t-test; [3]= Mann-Whitney U test; [4]= Chi-square test with Bonferroni correction

Supplementary Table 3: Demographics, lifestyle factors and comorbidity different H&Y stages vs controls

	H&Y 1	H&Y 2	H&Y 3	H&Y 4	Controls	P-value
n	15	33	12	10	31	
Female subjects	73.3%	39.4%	33.3%	40.0%	45.2%	0.210[1]
Mean Age	57.1 ± 10.7[a]	66.1 ± 8.0[b]	70.4 ± 8.6[b]	68.9 ± 9.0[b]	64.3 ± 8.9[ab]	0.002[2]
Mean Weight [kg]	66.7 ± 15.2	79.1 ± 14.7	81.4 ± 18.5	76.0 ± 9.8	77.8 ± 14.1	0.064[2]
Mean Height [cm]	165,5 ± 10.3	171.8 ± 10.2	175.4 ± 11.5	171.7 ± 8.1	170.1 ± 7.7	0.089[3]
**Lifestyle factors**						
Smoking	6.7%	0.0%	0.0%	0.0%	6.5%	0.540[1]
Coffee consumption > 2 cups per day	13.3%[a]	15.2%[a]	25.0%[ab]	30.0%[ab]	58.1%[b]	0.003[1]
Alcohol consumption ≥ twice a week	12.2%	36.7%	10.2%	6.1%	56.7%	0.520[1]
Probiotic supplement consumption	0.0%	6.1%	0.0%	10.0%	3.2%	0.217[1]
Prebiotic supplement consumption	0.0%	0.0%	0.0%	10.0%	3.2%	0.227[1]
Probiotic yogurt consumption ≥ once a week	0.0%	6.1%	8.3%	10.0%	0.0%	0.892[1]
Regular yogurt consumption ≥ once a week	57.1%	63.6%	41.7%	90.0%	61.3%	0.360[1]
Kefir or buttermilk consumption ≥ once a week	0.0%	6.1%	8.3%	0.0%	6.5%	0.868[1]
Banana consumption ≥ once a week	53.3%	63.6%	41.7%	60.0%	67.7%	0.595[1]
Asparagus consumption ≥ once a week	6.7%	9.1%	33.3%	0.0%	16.1%	0.413[1]
Chicory consumption ≥ once a week	0.0%	3.0%	16.7%	0.0%	16.1%	0.376[1]
Dandelion greens consumption ≥ once a week	0.0%	0.0%	0.0%	0.0%	0.0%	0.850[1]
Garlic consumption ≥ once a week	26.7%	33.3%	50.0%	40.0%	38.7%	0.870[1]
Artichoke consumption ≥ once a week	0.0%	0.0%	0.0%	0.0%	0.0%	NA
Leek consumption ≥ once a week	20.0%	12.1%	25.0%	10.0%	16.1%	0.546[1]
Onion consumption ≥ once a week	86.7%	78.8%	91.7%	80.0%	87.1%	0.377[1]
Wholemeal product consumption ≥ once a week	66.7%	72.7%	83.3%	80.0%	77.4%	0.798[1]
Adding salt to readily cooked dishes "often" or "always"	6.7%	12.1%	0.0%	0.0%	16.1%	0.678[1]

Easy physical activity [hours/week]	1.4 ± 1.4	1.6 ± 1.7	2.9 ± 3.4	2.3 ± 4.4	1.4 ± 1.7	0.736[3]
Moderate physical activity [hours/week]	2.2 ± 2.2	2.1 ± 2.3	1.7 ± 3.3	0.7 ± 1.3	2.5 ± 2.4	0.048[3]
Demanding physical activity [hours/week]	0.9 ± 2.6	0.1 ± 0.4	0.0	0.0	0.9 ± 3.3	0.043[3]
**Comorbidities**						
Mean NMS Score	5.3 ± 4.1	7.1 ± 4.2*	6.3 ± 5.5	11.3 ± 5.1*	2.7 ± 2.2	< 0.001[3]
NMS item "constipation"	20.0%[ab]	39.4%[a]	25.0%[ab]	40.0%[ab]	6.5%[b]	0.017[1]
NMS item "bowel emptying incomplete"	20.0%[ab]	18.2%[ab]	16.7%[ab]	50.0%[a]	6.5%[b]	0.049[1]
Degree of constipation symptoms (Wexner Score)	2.8 ± 3.3	3.9 ± 2.9	4.7 ± 4.8	6.5 ± 5.0	2.3 ± 1.9	0.051[3]
Degree of depression symptoms (GDS 30)	4.8 ± 5.5	6.5 ± 6.2	7.8 ± 5.6	11.0 ± 6.2*	4.3 ± 3.7	0.013[3]
Probable depression according to GDS 30	6.7%[ab]	9.1%[a]	25.0%[ab]	50.0%[b]	3.2%[a]	0.003[1]
Degree of REM sleep behaviour disorder symptoms (RBDSQ)	3.0 ± 3.1	4.4 ± 3.2*	6.5 ± 3.5*	6.2 ± 3.9*	1.4 ± 1.3	< 0.001[3]
Probable REM sleep behaviour disorder according to RBDSQ	33.3%[a]	45.5%[a]	66.7%[a]	70.0%[a]	0.0%[b]	< 0.001[1]
Restless Legs Syndrome	0.0%	6.1%	8.3%	20.0%	0.0%	0.074[1]
Dementia	0.0%	1.0%	0.0%	0.0%	0.0%	1.000[1]
Diabetes mellitus	0.0%	3.0%	0.0%	0.0%	3.2%	1.000[1]
Thyroid dysfunction (controlled)	20.0%	9.1%	8.3%	0.0%	9.7%	0.660[1]
Cholezystectomy	0.0%	3.0%	0.0%	10.0%	3.2%	0.640[1]
Appendectomy	0.0%	6.1%	0.0%	0.0%	0.0%	0.792[1]
Hysterectomy	0.0%	0.0%	0.0%	0.0%	3.2%	0.667[1]
Gynaecological Prolapse Repair	0.0%	3.0%	16.7%	10.0%	0.0%	0.062[1]
**Medication other than antiparkinsonian drugs**						
Betablocker	20.0%	18.2%	8.3%	20.0%	22.6%	0.904[1]
Selective Serotonine Reuptake Inhibitor	0.0%	20.0%	40.0%	40.0%	0.0%	0.017[1]
Acetylsalicylic Acid	0.0%	21.2%	8.3%	0.0%	9.7%	0.194[1]
Phenprocoumon	0.0%	3.0%	0.0%	0.0%	3.2%	1.000[1]
ACE-inhibitor or ATR-antagonist	13.3%	33.3%	25.0%	40.0%	32.3%	0.584[1]
Diuretic	6.7%	18.2%	8.3%	20.0%	22.6%	0.692[1]
Calcium Channel Blocker	0.0%	6.1%	8.3%	10.0%	16.1%	0.423[1]
Thyroxine	20.0%	12.1%	16.7%	0.0%	9.7%	0.646[1]
Antiepileptic	6.7%	6.1%	8.3%	20.0%	3.2%	0.388[1]

Statin	6.7%	15.2%	0.0%	30.0%	3.2%	0.086[1]
Laxatives	0.0%	6.1%	0.0%	10.0%	0.0%	0.325[1]
Tricyclic Antidepressants	0.0%	6.1%	0.0%	0.0%	0.0%	0.799[1]
Antihistaminics	0.0%	0.0%	0.0%	0.0%	6.5%	0.591[1]
Antiemetics	0.0%	3.0%	0.0%	0.0%	0.0%	1.000[1]
Protone pump inhibitors	0.0%[a]	12.1%[a]	0.0%[a]	40.0%[ab]	3.2%[c]	0.008[1]
Hormonal contraception in women	18.2%	0.0%	0.0%	0.0%	7.1%	0.432[1]
Postmenopausal hormone replacement in women	0.0%	7.7%	0.0%	0.0%	7.1%	1.000[1]

[1]= Fisher's exact test with Bonferroni correction; [2]= ANOVA with Bonferroni correction; [3]= Kruskal-Wallis test, * = vs. control $p < 0.0125$ Bonferroni corrected

Letters a, b and c indicate groups that do not differ on a 0.05 significance level.

Supplementary table 4:

Rank	Name	H+Y 1	H+Y 2	H+Y 3	H+Y 4
class	Betaproteobacteria	**0,016**	**0,019**	7,198	3,803
order	Burkholderiales	**0,016**	**0,019**	7,198	3,803
family	Lachnospiraceae	9,626	0,331	0,487	**0,030**
	Clostridiaceae	1,812	2,263	0,956	**0,011**
	Alcaligenaceae	**0,009**	**0,036**	9,220	6,073
genus	Faecalibacterium	1,137	1,769	1,673	**0,003**
	Sutterella	**0,009**	**0,036**	9,220	6,073

Supplementary table 5: Demographics, lifestyle factors and comorbidity PD vs controls (matched)

	Patients	Controls	P-value
**Demographics**			
n	28	19	
Female subjects	50.0%	52.6%	1.000[1]
Mean Age	62.6 ± 12.1	64.7 ± 10.7	0.556[2]
Mean Weight [kg]	76.3 ± 17.7	78.9 ± 15.6	0.610[2]
Mean Height [cm]	170.9 ± 11.6	169.7 ± 7.6	0.683[2]
**Lifestyle factors**			
Smoking	3.7%	10.5%	0.561[1]
Coffee consumption > 2 cups per day	32.1%	52.6%	0.228[1]
Alcohol consumption ≥ twice a week	53.6%	52.6%	1.000[1]
Probiotic supplement consumption	7.1%	0.0%	>0.05[4]
Prebiotic supplement consumption	0.0%	0.0%	>0.05[4]
Probiotic yogurt consumption ≥ once a week	7.7%	0.0%	>0.05[4]
Regular yogurt consumption ≥ once a week	55.6%	63.2%	>0.05[4]
Kefir or buttermilk consumption ≥ once a week	3.7%	11.8%	>0.05[4]
Banana consumption ≥ once a week	57.1%	63.2%	>0.05[4]
Asparagus consumption ≥ once a week	14.3%	15.8%	>0.05[4]
Chicory consumption ≥ once a week	3.7%	27.8%	>0.05[4]
Dandelion greens consumption ≥ once a week	0.0%	0.0%	>0.05[4]
Garlic consumption ≥ once a week	25.9%	42.1%	>0.05[4]
Artichoke consumption ≥ once a week	0.0%	0.0%	>0.05[4]
Leek consumption ≥ once a week	17.9%	5.6%	>0.05[4]
Onion consumption ≥ once a week	85.7%	88.9%	>0.05[4]
Wholemeal product consumption ≥ once a week	73.1%	63.2%	>0.05[4]
Adding salt to readily cooked dishes "often" or "always"	7.1%	15.8%	>0.05[4]
Easy physical activity [hours/week]	1.5 ± 1.1	1.1 ± 1.4	0.125[3]
Moderate physical activity [hours/week]	1.5 ± 1.6	2.1 ± 1.7	0.234[3]
Demanding physical activity [hours/week]	0.0 ± 0.2	1.1 ± 4.1	0.209[3]
**Comorbidities**			
Mean NMS Score	3.6 ± 3.0	3.4 ± 2.3	0.879[3]
NMS item "constipation"	10.7%	10.6%	1.000[1]
NMS item "bowel emptying incomplete"	14.3%	5.3%	0.635[1]
Degree of constipation symptoms (Wexner Score)	2.5 ± 2.7	2.4 ± 2.3	0.996[3]
Degree of depression symptoms (GDS 30)	4.3 ± 3.9	4.8 ± 4.3	0.758[3]
Probable depression according to GDS 30	7.1%	5.3%	1.000[1]
Degree of REM sleep behaviour disorder symptoms (RBDSQ)	3.1 ± 2.9	1.8 ± 1.4	0,289[3]
Probable REM sleep behaviour disorder according to RBDSQ	28.6%	0.0%	0.015[1]
Restless Legs Syndrome	0.0%	0.0%	
Dementia	0.0%	0.0%	
Diabetes mellitus	3.6%	0.0%	1.000[1]
Thyroid dysfunction (controlled)	14.3%	5.3%	0.635[1]

Cholezystectomy	0.0%	5.3%	0.404[1]
Appendectomy	0.0%	0.0%	
Hysterectomy	0.0%	0.0%	
Gynaecological Prolapse Repair	0.0%	0.0%	
**Medication**			
L-Dopa	50.0%	0.0%	<0.001[1]
COMT Inhibitor	7.1%	0.0%	0.508[1]
Dopamine Agonist	82.1%	0.0%	<0.001[1]
MAO-B Inhibitor	64.3%	0.0%	<0.001[1]
Amantadine	14.3%	0.0%	0.137[1]
Anticholinergic	0.0%	5.3%	0.404[1]
Betablocker	25.0%	21.1%	1.000[1]
Selective Serotonine Reuptake Inhibitor	3.6%	0.0%	1.000[1]
Acetylsalicylic Acid	10.7%	5.3%	0.638[1]
Phenprocoumon	3.6%	5.3%	1.000[1]
ACE-inhibitor or ATR-antagonist	28.6%	26.3%	1.000[1]
Diuretic	7.1%	21.1%	0.204[1]
Calcium Channel Blocker	7.1%	10.5%	1.000[1]
Thyroxine	17.9%	5.3%	0.378[1]
Antiepileptic	3.6%	0.0%	1.000[1]
Statin	7.1%	0.0%	0.508[1]
Laxatives	0.0%	0.0%	
Tricyclic Antidepressants	3.6%	0.0%	1.000[1]
Antihistaminics	0.0%	5.3%	0.404[1]
Antiemetics	0.0%	0.0%	
Protone pump inhibitors	0.0%	0.0%	
Hormonal contraception in women	14.3%	10.0%	>0.05[4]
Postmenopausal hormone replacement in women	0.0%	10.0%	0.417[1]

[1]= Fisher's exact test; [2]= t-test; [3]= Mann-Whitney U test; [4]= Chi-square test with Bonferroni correction
