# Supplementary material for: Changes in Gastrointestinal Microbiome Composition in PD: A Pivotal Role of Covariates
Source: Front Neurol. 2020 Sep 23;11:1041. doi: 10.3389/fneur.2020.01041 (PMC7538808; doi:10.3389/fneur.2020.01041)
Supplement: Supplementary file 3 [file Data_Sheet_3.PDF]

Date: _______________

Height: ______ cm **Weight:**_____ kg

Age: _____ Jahre **Gender:** □ f □ m

1. **Do any of your family members suffer from Parkinson's disease?**
   □ Yes … If yes, who? ____ □ No

2. **Do you have a HIV infection?**
   □ Yes □ No

3. **Has a doctor diagnosed you with a persistent disease of the gastrointestinal tract ?**
   □ Yes □ No
   If yes, which one?
   □ Celiac disease
   □ Pernicious anemia
   □ Autoimmune gastritis
   □ Symptomatic diverticulosis
   □ Inflammatory bowel disease
   □ Irritable bowel syndrome
   □ Strictures
   □ Adhesions
   □ Varicosis or diverticulum of the esophagus
   □ Meckel's diverticulum
   □ Other (if possible, provide exact medical term):
   ____

4. **Has a doctor diagnosed you with a hormonal disease?**
   □ Yes □ No
   If yes, which one?
   □ Diabetes mellitus
   □ Thyroid dysfunction: Which one? _____________________
   □ Other (if possible, provide exact medical term):
   ____

5. **Have you been diagnosed with an active or previous hepatobiliar or pancreatic disease?**
   □ Yes … If yes, which one? ____
   □ No

6. **Have you undergone previous abdominal or anorectal surgery?**
   □ Yes… If yes, which kind of surgery? __________________________________ When?
   ____
   □ No

7. **Women only: Have you been diagnosed with severe gynaecological prolapse ?**
   □ Yes … If yes, have you undergone a repair procedure? □ Yes □ No
   If yes, when? _________________
   □ No

8. **Have you been diagnosed with any of the following:**

Peritonitis	□ Yes	□ No
Polyneuropathy	□ Yes	□ No
Polio	□ Yes	□ No
Spina bifida	□ Yes	□ No
Paraparesis	□ Yes	□ No
Symptomatic peripheral arteriosclerosis	□ Yes	□ No
Intestinal ischemia	□ Yes	□ No
Aortic aneurysm or dissection	□ Yes	□ No
Connective tissue disease	□ Yes	□ No
Autoimmune disease	□ Yes… if yes, which one? ______________________	□ No
Sarcoidosis outside of lung and skin	□ Yes	□ No
Active cancer	□ Yes	□ No
Abdominal, intestinal, or urogenital fistula	□ Yes	□ No
Severe renal insufficiency	□ Yes	□ No
Women only: Severe endometriosis	□ Yes	□ No

9. **Have you been diagnosed with severe spinal stenosis within the last 12 months?**
   □ Yes □ No

10. **Have you been diagnosed with any of the following within the last two months?**

Severe hypokalemia or hyperkalemia which required hospital treatment	□ Yes	□ No
Abdominal trauma	□ Yes	□ No
Gastrointestinal tract infection	□ Yes	□ No
Food intoxication	□ Yes	□ No
Major epistaxis which required medical treatment	□ Yes	□ No

11. **Have you undergone any of the following procedures within the last two months?**

Narcosis or analgosedation	□ Yes	□ No
Endoscopic procedure of the gastrointestinal tract	□ Yes	□ No

12. **Have you been diagnosed with any oft he following?**

Restless legs syndrome	□ Yes	□ No
Psychosis	□ Yes	□ No
Dementia	□ Yes	□ No
Alcohol abuse	□ Yes	□ No
Drug abuse	□ Yes	□ No

13. **Have you taken any antibiotics within the last month?**
    □ Yes… If yes, which one? ________________________________ □ No

14. **Women only: Do you use any hormonal contraception or did you use one in the past?**
    □ Yes, I currently use hormonal contraception. Which one? ____________________
    □ No, I currently do not use any hormonal contraception but did use hormonal contraception in the past. Which one? ____
    □ No, I have never used any hormonal contraception.

15. Women only: Do you take postmenopausal hormone replacement prescribed by your doctor?

□ Yes □No

16. Do you regularly take any medication?

□ Yes … If yes, please precise in the following table. □ No

Name of medication	Dosage	Time points of medication intake per day

17. How much coffee do you usually drink?

□ Less than one cup per day
□ 1-2 cups per day
□ 2-6 cups per day
□ More than 6 cups per day

18. Do you smoke?

□ Yes… If yes, for how many years? _____________ Hoy many cigarettes per day? ___________
□ No, I used to smoke in the past but quit. When? __________________
□ No, never

19. How often do you drink alcohol?

□ never
□ approximately once a month
□ 2-4 times a month
□ 2-3 times a week
□ 4 times or more a week

20. How often do you take the following dietary supplements?

	daily	4-6 times per week	1-3 times per week	1-3 times per month	1-2 times in three month or less	Never	Do not know
Probiotic dietary supplements (e.g. capsules or powder)	☐	☐	☐	☐	☐	☐	☐
Prebiotic dietary supplements (e.g. capsules or powder)	☐	☐	☐	☐	☐	☐	☐

21. How often do you eat the following foods?

	daily	4-6 times per week	1-3 times per week	1-3 times per month	1-2 times in three month or less	Never	Do not know
Probiotic yogurt	☐	☐	☐	☐	☐	☐	☐
„normal“ yogurt not being declared „probiotic“	☐	☐	☐	☐	☐	☐	☐
Kefir or buttermilk	☐	☐	☐	☐	☐	☐	☐
Bananas	☐	☐	☐	☐	☐	☐	☐
Asparagus	☐	☐	☐	☐	☐	☐	☐
Chicory	☐	☐	☐	☐	☐	☐	☐
Dandelion greens	☐	☐	☐	☐	☐	☐	☐
Garlic	☐	☐	☐	☐	☐	☐	☐
Artichokes	☐	☐	☐	☐	☐	☐	☐
Leeks	☐	☐	☐	☐	☐	☐	☐
Onions	☐	☐	☐	☐	☐	☐	☐
Wholemeal products (wheat, barley, rye)	☐	☐	☐	☐	☐	☐	☐

22. How often do you add more salt to readily cooked dishes?

□ (Almost) always

□ Often

□ Rarely

□ Never

23. How much time to you spend with the following activities per week?

	**Hours per week**
**Easy** physical activity (e.g. bowling, yoga)	_______
**Moderate** physical activity (e.g. hiking, bicycling, tennis, nordic walking, skiing, swimming)	_______
**Demanding** physical activity (e.g. jogging, soccer, handball, mountaineering, athletics, mountainbiking)	_______

Sources:

Scheperjans et al. Gut Microbiota Are Related to Parkinson's Disease and Clinical Phenotype. Mov Disord 2015; 30: 350-358

Bundesministerium für Verbraucherschutz, Ernährung und Landwirtschaft. Fragebogen Nationale Verzehrstudie II. „Was esse ich“ [online im Internet]. https://www.mri.bund.de/fileadmin/MRI/Institute/EV/NVS_II_Fragebogen.pdf [Stand: 19.08.2016].

Moshfegh A, Friday JE, Goldman JP et al. Presence of Inulin and Oligofructose in the Diets of Americans. J Nutr. 1999; 129: 1407S-11S
